# Tumor beta2-microglobulin and HLA-A expression is increased by immunotherapy and can predict response to CIT in association with other biomarkers

**DOI:** 10.3389/fimmu.2024.1285049

**Published:** 2024-02-22

**Authors:** Bernhard Reis, Jan Attig, Sebastian Dziadek, Nico Graefe, Astrid Heller, Natascha Rieder, Bruno Gomes

**Affiliations:** ^1^ Roche Pharmaceutical Research and Early Development Oncology, Roche Innovation Center Basel, Basel, Switzerland; ^2^ Roche Pharmaceutical Research and Early Development, Pharmaceutical Sciences - Biomarkers, Bioinformatics and Omics & Pathology, Roche Innovation Center Basel, Basel, Switzerland; ^3^ Roche Pharmaceutical Research and Early Development Oncology, Roche Innovation Center Munich, Penzberg, Germany

**Keywords:** checkpoint inhibitor therapy, major histocompatibility complex class I, beta2microglobulin, immunotherapy, immunohistochemistry, human leukocyte antigen-A

## Abstract

**Background:**

Downregulation of MHC class I expression and/or defects in the antigen presentation pathways are commonly reported in human cancers. Numerous studies previously have explored extensively the molecular mechanisms that underlie HLA-class I and Beta2-Microglobulin (B2M) downregulation. However, the techniques presently available to detect expression of MHC class I proteins lack the robustness, specificity and sensitivity needed for systematic integration and analysis in clinical trials. Furthermore, the dynamics of HLA-class I and B2M expression have not been comprehensively studied as a potential biomarker for immunotherapy.

**Methods:**

Using novel, validated, immunohistochemistry (IHC)-based methods for quantifying B2M and HLA-A in tumor samples from diverse cancer types, we have determined loss of B2M and HLA-A proteins in 336 archived, primary specimens and 329 biopsies from metastatic patients collected during Roche-sponsored Phase 1 clinical trials investigating novel immunotherapy candidates as monotherapy or in combination with CPI.

**Results:**

Up to 56% of cases with B2M or HLA-A loss were noted in the investigated tumor types. The frequency of loss was dependent on indication and stage of disease and revealed heterogeneous expression patterns across patients. B2M and HLA-A loss was increased in metastatic lesions compared to primary tumors, indicating selection of MHC class I low clones in metastatic and refractory tumor cells. High on-treatment B2M expression correlated with successful clinical outcome (RECIST), while high baseline B2M did not. A treatment-induced increase of B2M expression was noted in most of the patients with low B2M levels at baseline. The triple biomarker combination of B2M, CD8 and PDL1 strongly improved response prediction to cancer immunotherapy.

**Conclusion:**

Our results indicate that B2M and HLA-A loss occurs frequently in tumors and is reversed in most instances following immunotherapy which supports the conclusion that MHC class I loss is not the dominant resistance mechanism to CPI treatment. This investigation reveals a highly dynamic expression of HLA-A and B2M in tumors affected by indication, metastatic status, immunophenotype and immunotherapy treatment. Baseline expression levels of B2M on tumors may be of utility as a constituent of a biomarker panel used for selecting patients for immunotherapy clinical trials.

## Introduction

The discovery of checkpoint inhibitors (CPI) like CTLA-4 and PD-1 and subsequently drugs targeting these molecules have revolutionized cancer therapy (1). The central tenet of cancer immunotherapy is that elements of the immune system, specifically CD8+ T cells, recognize and destroy tumor cells (2). CD8+ T cells typically identify cancer cells by recognizing tumor-associated antigens presented on the cell surface in conjunction with MHC class I antigen (3). It is therefore postulated that under pressure exerted by the immune system, tumor cell variants that lack expression of MHC class I arise by a process called ‘immunoediting’ (4). Downregulation of MHC class I expression and its constituent components is thought to be an important mechanism by which tumors escape immune-mediated destruction, potentially representing a significant challenge to immunotherapy (5, 6).

Beta2-Microglobulin (B2M) is a 12 kda protein made up of 119 amino acids that is encoded by a gene located on chromosome 15. It is an essential component of MHC class I molecules and provides structural integrity and stability to the MHC class I-peptide complex (7). It is expressed by virtually all nucleated cells. B2M mutations are known to occur with varying frequencies in various tumor types (8) and some tumors like bladder cancer are known to downregulate B2M in the absence of hard mutations within the gene (9). Downregulation of B2M is generally believed to facilitate escape of tumors from immunosurveillance and immune-mediated destruction (10, 11), since loss of B2M is typically associated with reduced MHC I expression. However, this point of view is not incontrovertible. Some studies with human tumor specimens and animal models have demonstrated that downregulation of B2M and HLA give rise to tumors with poor prognosis and resistance to CPI therapy (12, 13). Other studies, however, have reported that B2M and HLA-I loss are associated with better prognosis and prolonged survival (14–16). Dissimilarities in immunohistochemistry techniques, antibody clones and quantification methods between studies may have contributed to discrepancies in reported frequencies and intensities of B2M and MHC class I expression.

The primary focus of the present study was to examine whether B2M and HLA expression can be used as a practical biomarker for immunotherapy studies without emphasis on the underlying molecular mechanism. For this, we have developed robust, reproducible assays for IHC staining and quantification of B2M and HLA-A expression on tumor cells. We have then used these assays to analyze the expression of B2M and HLA-A in diverse, primary, and metastatic tumors including longitudinal samples acquired before start and during treatment with various immunotherapy drug candidates. The expression analysis results were correlated to other biomarkers and clinical outcomes.

## Materials and methods

### Tumor samples

Primary tumor specimens collected during routine patient management were utilized to validate B2M and HLA-A assays and to establish prevalence in the following disease indications with the numbers of stained cases given in brackets: breast cancer (n=46), cervical cancer (n=16), colorectal cancer (n=54), esophageal cancer (n=9), gastric cancer (n=20), head and neck cancer (n=59), hepatocellular cancer (n=18), melanoma (n=64), non-small cell lung cancer (n=67), renal cell cancer (n=21), urinary bladder cancer (n=31) and small cell lung cancer (n=15).

### Clinical samples

**Figure 1:**
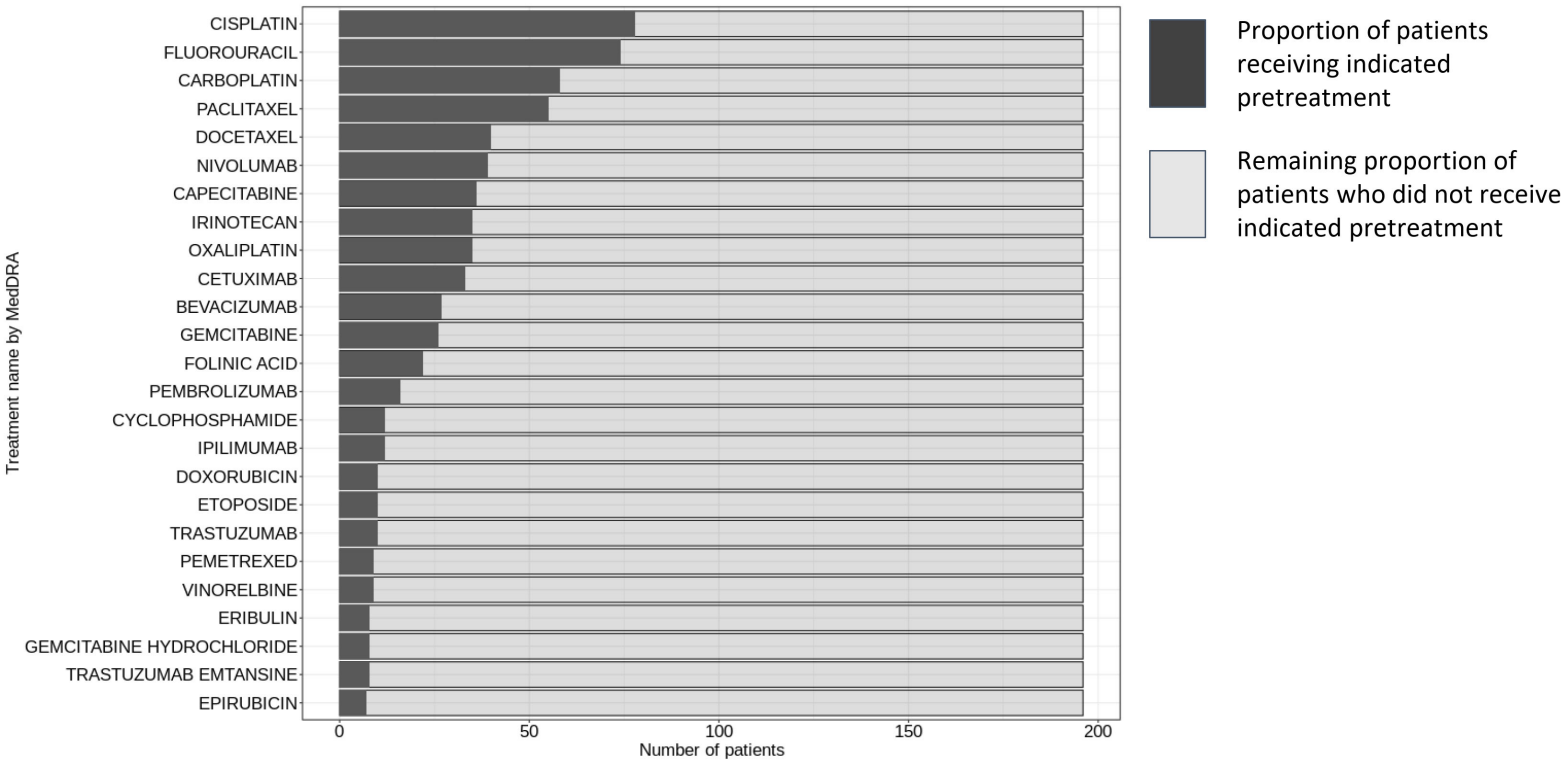
Pre-treatment regimens in patient cohort for HLA-A and B2M staining evaluation. This figure displays the distribution of pre-treatment regimens administered to the 213 patients prior to initiating the study’s trial protocol. The data was sourced from Clinical Study Reports by Roche, with the inclusion criteria limited to treatments received (i) before the first dose within the scope of the trial protocol, and (ii) designated either as premedication or as cancer therapy. The nomenclature for the treatments adheres to the conventions established by the Global Drug Safety Reporting (GDSR) system and the Medical Dictionary for Regulatory Activities (MedDRA).

Clinical specimens consisting of 213 baseline and 116 on-treatment biopsies from metastatic lesions were collected from patients enrolled in several phase I clinical trials. The agents under investigation included simlukafusp alfa (FAP-IL2v), cergutuzumab (CEA-IL2v), and lirilumab (anti-CD40), targeting various solid tumors. The majority of the patients, approximately 75%, were also treated with a combination therapy that included either atezolizumab or pembrolizumab. Additional combination treatments administered according to the study protocols in a subset of patients comprised trastuzumab, vanucizumab, and bevacizumab. Details of the clinical studies, patient numbers, CPI combination treatments and patient demographics are provided in **Tables 1**, **2**. **Figure 1** presents an overview of prior treatments, while **Supplementary Figure 1** offers a detailed breakdown of these treatments for each study.

**Table 1:** Sample and clinical study overview.

Ct.gov identifier, Study ID and other reference	Phase and patient population	Study drug(s)	Total number of samples (number of patients receiving CPI combination treatment)	
			Screening	On treatment
NCT02304393, BP29392 Barlesi et al., 2020	Phase Ib, advanced solid tumors	Selicrelumab (anti-PD-L1; RO7009789) in combination with atezolizumab	39 (37)	32 (31)
NCT02350673, BP29435, Van Brummelen et al., 2018	Phase Ib, locally advanced and/or metastatic carcinoembryonic antigen (CEA)-positive solid tumors	Cergutuzumab amunaleukin (CEA-IL2v, RO6895882) in combination with atezolizumab	12 (12)	5 (5)
NCT02627274, BP29842, Melero et al., 2018	Phase Ia/Ib, metastatic solid tumors	Simlukafusp alfa (FAP IL2v, RO6874281) as a single agent or in combination with trastuzumab or cetuximab	24 (0)	12 (0)
NCT02665416, BP29889	Phase Ib, metastatic solid tumors	Selicrelumab (RO7009789) with vanucizumab or bevacizumab	28 (0)	15 (0)
NCT03063762, BP39365	Phase Ib, unresectable advanced and/or metastatic renal cell carcinoma (RCC)	Simlukafusp alfa (FAP IL2v, RO6874281) in combination with atezolizumab with/without bevacizumab	20 (18)	16 (15)
NCT03386721, BP40234, Italiano et al., 2021	Phase II, advanced and/or metastatic solid tumors	Simlukafusp alfa (FAP IL2v, RO6874281) in combination with atezolizumab	63 (60)	37 (37)
NCT03875079, BP41054	Phase Ib, advanced or metastatic melanoma	Simlukafusp alfa (FAP IL2v, RO6874281) in combination with pembrolizumab	26 (26)	0

This table lists the metastatic biopsy samples derived from Phase I Roche clinical trials which were used in this study, the associated Roche-sponsored clinical trials and treatments investigated, and the respective number of patients receiving combination treatment with CPI (either Atezolizumab or Pembrolizumab).

**Table 2:** Patient demographics and cohort characteristics.

		Pts with biopsy at pre-study Screening phase (n= 230)	PTs with biopsy on treatment (n = 117)
Age	median (min - max)	58 (21 - 86)	58 (23 - 81)
Sex	male female	118 (52%) 111 (48%)	59 (46%) 50 (54%)
ECOG	0	99 (43.0%)	46 (42.2%)
	1	129 (56.1%)	62 (56.9%)
	missing	2 (0.9%)	1 (0.9%)
Lines of treatment	1	35 (16%)	10 (9.2%)
	2-3	86 (38%)	39 (35.8%)
	4-5	47 (20%)	27 (24.8%)
	6-9	22 (10%)	11 (10.1%)
	> 9 (max 17)	6 (2%)	5 (4.6%)
	missing	34 (14%)	17 (15.6%)
# of metastasis at screening	0	18 (8%)	10 (9.2%)
	1-3	129 (56%)	67 (61.5%)
	5-8	59 (26%)	26 (23.9%)
	> 8	23 (10%)	6 (5.5%)
	missing	1 (0%)	0 (0%)
CPI pre-treatment (all approved anti PD1, PD-L1 or CTLA-4 therapies)	yes	58 (26%)	22 (20.2%)
	no	164 (72%)	84 (77.1%)
	un- documented	8 (4%)	3 (2.8%)
Previous systemic cancer therapy, n (%)	No	35 (15.2%)	18 (16.5%)
	Yes	195 (84.8%)	91 (83.5%)
previous Radiotherapy	No	112 (48%)	52 (47.7%)
	Yes	118 (52%)	57 (52.3%)

This table lists patient demographics and cohort characteristics of the patients giving the metastatic biopsy samples as part of their participation in Phase I clinical trials sponsored by Roche.

Baseline biopsies were collected during the screening period to assess patient eligibility for the trial, typically ranging from one day to three weeks prior to the commencement of the investigational treatment.

Biopsy samples taken during treatment were collected at the expected peak of intratumoral pharmacodynamic effects for the specific treatment being investigated. These maximum effects are generally anticipated after one to four treatment cycles. The treatment schedule varied depending on the administered drug, ranging from once weekly to once every three weeks. The timing for collecting these on-treatment biopsies was strictly regulated, adhering to the detailed guidelines outlined in each respective clinical protocol.

### Immunohistochemistry

For the development of B2M and HLA assays, a comprehensive series of antibodies and staining parameters were evaluated to establish optimal antibody reagents and conditions. These conditions were tailored to facilitate the detection of the target antigens with maximal specificity and sensitivity. The specificity and sensitivity of the analytes were rigorously tested during the immunohistochemistry (IHC) assay development phase.

The assessment utilized the Tour of Tumor (TOT) and Tour of Body (TOB) BC9 microarray (provided by SuperBioChips Laboratories, Seoul, Korea), which comprises a diverse array of 30 normal human tissue types and 29 human tumor types. This microarray was instrumental in appraising the precise staining and sub-cellular localization of the HLA-A EP1395Y clone, while also monitoring for potential off-target staining.

The HLA-A localization results were consistent with expectations in both TOB and TOT tissues, predominantly marking the membrane and cytoplasm. Staining patterns observed in normal tissue corresponded with data published previously, noting low expression levels in brain, pancreas, and muscle tissues—a pattern that was mirrored in both HLA-A and B2M staining.

Specific experiments to assess the binding pattern with respect to HLA-A alleles and potential off-target reactivity against other HLA-I alleles were not conducted. The antibody used was sourced from Abcam. For immunization, a synthetic peptide within the 50-150 amino acid range of Human HLA-A was utilized, the details of which remain proprietary.

After an initial screening to estimate primary protocols, the final assays were generated by refining and optimizing the combination of conditions that produced the most reliable and robust assays. The assay parameters optimized included screening antibody diluents, epitope unmasking, and titration of the antibodies. The background (nonspecific staining) of the assays was also examined at this step, as were the integrity of cell and tissue morphology, and the appearance of proper cell type and cell localization. Prior to staining of any patient tissue, the performance specifications for accuracy, precision, analytical sensitivity, analytical specificity, long term reagent and tissue section stability and any other applicable performance characteristics were established for each of the developed tests according to 42 CFR § 493.1253 (https://www.govinfo.gov/app/details/CFR-2013-title42-vol5/CFR-2013-title42-vol5-sec493-1253).

Immunohistochemistry was performed on formalin-fixed and paraffin-embedded resection specimens or biopsies, sectioned at 4µm thickness (3µm for CD8). Details of the staining procedure are as follows:

#### B2M IHC

Instrument - Benchmark Ultra (Roche Diagnostics AG, Switzerland); Antigen Retrieval - CC1 for 64 minutes; Antibody Rabbit Monoclonal - SP433 (Roche- generated by immunization of rabbits with peptide Aa 100-119, located in the C-terminal end (CRVNHVTLSQPKIVKWDRDM), Stock Conc. - 0.9 mg/ml, Working Conc. - 1/15000 (0.06 µg/ml); Diluent - 90103, Incubation Time - 16 minutes, Incubation Temp. - 36°C; Negative Reagent Control - Rabbit monoclonal (790-4795); Detection - OptiView.

#### HLA-A IHC

Instrument - Benchmark Ultra; Antigen Retrieval - CC1 for 64 minutes; Antibody Rabbit Monoclonal - clone EP1395Y (Abcam; Cambridge, UK; ab52922), Stock Conc. - 0.2 mg/ml, Working Conc. - 1/500 (0.4 µg/ml); Diluent - 90103, Incubation Time - 16 minutes, Incubation Temp. - 36°C; Negative Reagent Control - Rabbit monoclonal (790-4795). Detection - OptiView.

#### Ki67/CD8 IHC

instrument - Discovery Ultra (Roche Diagnostics, Switzerland); Antigen Retrieval - CC1 for 32 minutes; Primary antibodies: rabbit monoclonal CD8 clone SP239 (Abcam ab178089) at 5 µg/ml for 32 mins; rabbit monoclonal Ki67 antibody (30-9, RTD catalog 790-4286, Ready-to-use) incubated for 8 mins. Detection: Discovery yellow Kit (RTD. 760-239; Roche Diagnostics, Switzerland) for CD8 and Discovery Purple Kit (RTD. 760-229) for Ki-67.

#### PD-L1 IHC

commercially purchased VENTANA PD-L1 (SP142) Assay & VENTANA PD-L1 (SP263) Assay were used according to the manufacturer’s instructions.

### Analysis and quantification of staining by light microscopy

B2M, HLA-A and PD-L1 IHC slides were visually assessed by certified pathologists.

#### B2M/HLA-A

B2M and HLA-A expression on tumor cells was assessed based on morphological features without co-staining with a tumor cell marker. For each specimen, the percentage of B2M and HLA-A positive tumor cells (irrespective of the staining intensity) was separately documented.

#### Tumor PD-L1 expression

Diagnostic assays SP142 and SP263 with respective sensitivity cut-offs were used to label tumors as PDL1+ and PDL1-. The percentage of PD-L1 positive tumor cells relative to all tumor cells (TC) and the percentage of tumor area occupied by tumor infiltrating PD-L1 positive immune cells (IC) were estimated for each specimen, irrespective of the type of PD-L1 assay used.

#### CD8+

The density of total CD8+ cells in the tumor area were determined using a digital algorithm on digital images of Ki67/CD8 stained slides. In addition, immunophenotyping was performed on those images by a pathologist. The abundance of CD8+ cells was separately assessed in tumor cell nests (i.e. tumor epithelium; IE) and tumor stroma (ITS). To capture the heterogeneity, the percentage of area covering very low (0), low (1), moderate (2) or high (3) amounts of CD8+ cells was estimated per compartment. A threshold of 20% area coverage for IE and ITS at abundance 2 + 3 was used to define the immunophenotype (17, 18) according to the following rules: Deserted:<20% IE 2 + 3 and<20% ITS 2 + 3; inflamed: ≥20% IE 2 + 3; excluded:<20% IE 2 + 3 and ≥20% ITS 2 + 3.

### Data analysis and statistics

#### Defining cut-off to binarize samples into HLA-A/B2M positive and negative samples

The type of tumor had a clear and significant impact on frequency of positive cells for both B2M and HLA-A (p value< 10^-7^ in both cases, ANOVA). In tumor types with sufficient sample sizes (>30), primary tumor samples showed a bi-modal distribution; a mixture model estimated the mean for the two distributions at 3.6% and 97% for B2M (for HLA-A 6% and 97%, respectively). Hence, we chose 50% cut-off as close to the optimal theoretical cut-off while also giving an intuitive clinical and biological interpretation.

#### Significance testing

Using the 50% cut-off criterion, significance testing of differences in proportion of tumors scored as HLA-A and B2M- positive or negative were done using **χ^2^**-test for equal proportion. Significance testing of differences in the amount of B2M and HLA-A-positive staining cells was done using logistic regression models, testing the likelihood of B2M/HLA-A as independent variables. To test for significant association between PDL1 positive staining and likelihood for HLA-A or B2M positive cells, a covariate was added to account for the two PDL1 antibodies between samples (e.g.


$$\text{ln}(\frac{percent\;PDL1+}{1-percent\;PDL1+})˜\text{ln}(\frac{percent\;B2M+}{1-percent\;B2M+})+antibody^{clone}$$

Significance tests were performed with the *stats* package implementations in R, versions 4.01 to 4.2.0 (19). The meta-regressions were done using the metagen package (20).

### Receiver-operating-curve analysis

For receiver-operating-curve analysis, first univariate logistic regression models were tested. CD8 density showed a clear non-linear relationship with response which was substantiated by independent data within Roche (unpublished data). For this reason, CD8 density was fit using natural cubic splines with 2 degrees of freedom, or 3 classes split at 112.5 and 411.8 cells/mm2 (knots). All other predictors were fit as linear predictors.

## Results

Three antibodies reactive to B2M were tested with our IHC protocol: EPR21752-214 (Abcam, rabbit monoclonal antibody), B2M-01 (Invitrogen/Abcam, mouse monoclonal antibody) and SP433, a rabbit monoclonal antibody generated by Roche. All 3 clones were tested for staining characteristics. Of these antibodies, SP433 was consistently superior in comparison to EPR21752-214 and B2M-01 (data not shown). Therefore, SP433 was selected for B2M detection.

**Figure 2:**
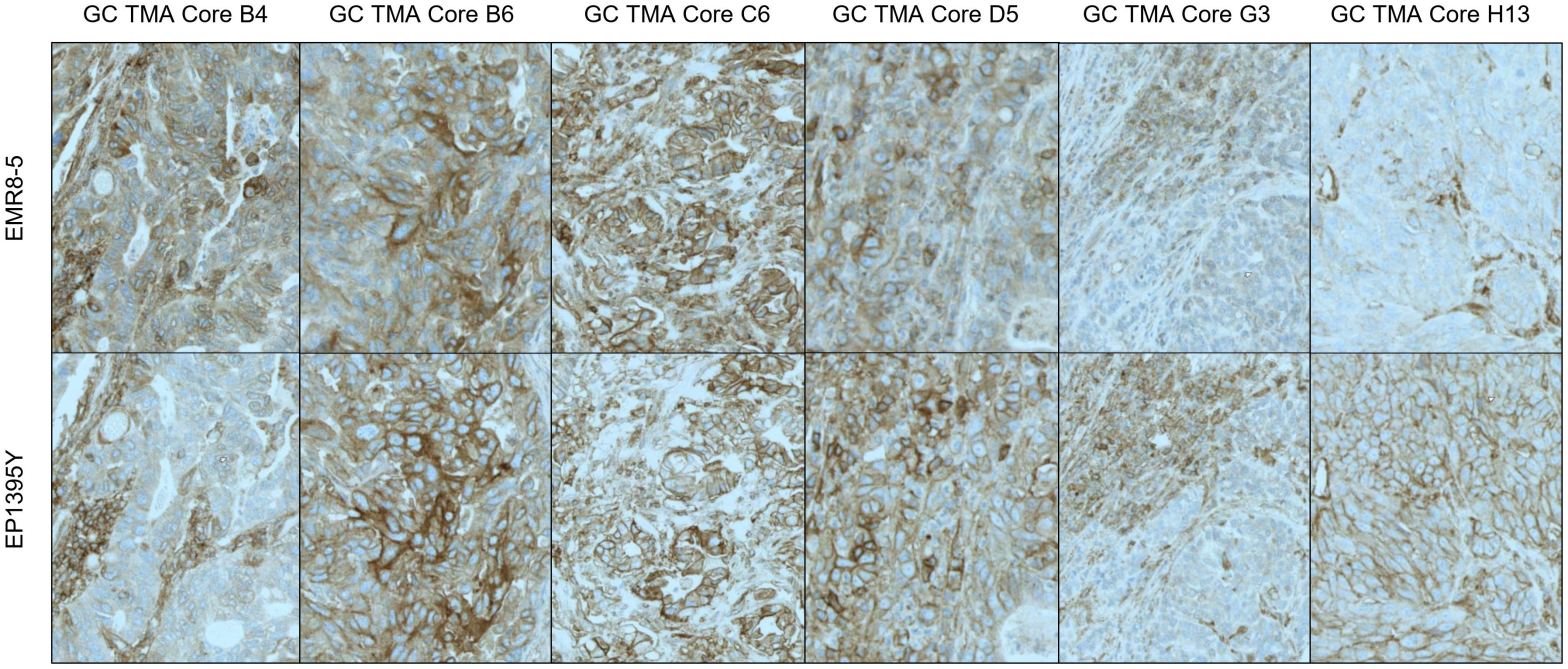
Pan-HLA-ABC and HLA-A - Differential Staining Pattern EMR8-5 vs EP1395y antibody clones. Antibody clone EMR8-5 is a pan HLA-ABC recognizing antibody, EP1395Y is HLA-A specific. The performance of both antibodies was compared using tissue microarrays. In most cores, EP1395Y shows a more distinct and cleaner staining pattern than EMR8-5.

Primary antibodies tested for IHC staining of HLA included the mouse monoclonals EMR8-5 (Abcam) and HCA2 (Acris/Origene), reactive to HLA-ABC heavy chain and EP1395Y (Abcam, rabbit monoclonal) which recognizes only HLA-A. Antibody clone W6/32, an antibody clone recognizing HLA-A, B and C used in several studies (21) had to be excluded, as this antibody recognizes a conformational epitope and therefore was not suitable for paraffin embedded samples. HCA-2 did not meet the performance criteria, as it was sensitive to age of the tissue paraffin blocks (data not shown). The two remaining clones EMR8-5 (specific to HLA-A, B and C) and EP1395Y (specific to HLA-A) performed comparably (**Figure 2**). However, EP1395Y was superior to EMR8-5 in detecting membrane staining, while EMR8-5 signal was frequently localized to the cytoplasm and had an indistinct appearance which suggested nonspecific reactivity. Therefore, EP3195Y was selected for further method qualification and prevalence assessment. Consequently, in this study, HLA-A was used as a putative biomarker and surrogate for HLA-A, B or C, acknowledging the limitation that detection of HLA-A loss does not exclude the possibility of presence of HLA-B or C. However, the suitability of using HLA-A expression with acceptable sensitivity to indicate loss of all three HLA class I proteins is substantiated by several reports showing frequent concurrent loss of expression of HLA-A, B and C (21, 22). Overall, EMR8-5 and EP3195Y gave consistent results as can be seen by a representative comparison of parallel staining shown in (**Figure 2**). Surprisingly, EP3195Y reactive to HLA-A alone was even more sensitive in detecting MHC I expression compared to EMR8-5 which was pan-HLA reactive (core H13 in **Figure 2**).

**Figure 3:**
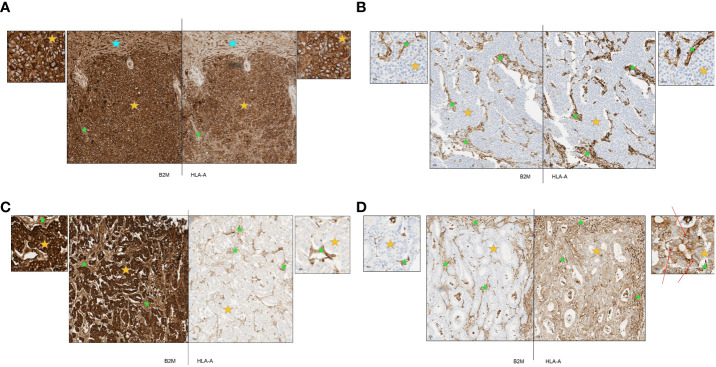
**(A-D)** Representative primary tumors stained by immunohistochemistry for B2M and HLA-A. Orange stars depict tumor tissue; green stars depict stroma and blue stars depict normal tissue. **(A)** Representative case of hepatocellular carcinoma with strong expression of B2M and HLA-A. B2M and HLA-A show a similar pattern of expression. All tumor cells were strongly positive and normal liver tissue and tumor stroma were also positive. **(B)** Representative case of urothelial carcinoma with complete loss of B2M and HLA-A expression. B2M and HLA-A show a similar pattern of expression. All tumor cells were negative with positive staining in tumor stroma. **(C)** Representative case of esophageal carcinoma with strong expression of B2M and complete loss of HLA-A. All tumor cells stained strongly positive for B2M with positive staining in tumor stroma and blood vessels. Tumor cells were negative for HLA-A expression although tumor stroma and blood vessels were positive. **(D)** Representative case of colorectal cancer with complete loss of B2M and moderate expression of HLA-A. All tumor cells were negative for B2M while stromal areas were positive. Tumor cells displayed cytoplasmic staining for HLA-A while some membrane staining was present (red arrows); positive staining in tumor stroma.

After establishment of robust and reproducible IHC assays for B2M and HLA-A, we utilized these to examine the expression of B2M and HLA-A on tumor cells of diverse cancer types. Representative staining patterns are provided in **Figure 3**. **Figure 3A** is an example of a hepatocellular carcinoma displaying strong expression on both tumor and stroma as well as normal liver tissue (marked by orange, green and blue stars respectively). **Figure 3B** shows complete loss of expression of both proteins on urothelial carcinoma tumor cells (orange star) with clear and distinct positive staining of stroma (green star). Presence or absence of B2M and HLA-A did not necessarily coincide in all the specimens tested. To illustrate this, we present an esophageal carcinoma specimen with loss of HLA-A and high expression of B2M (**Figure 3C**). In contrast, an example of colorectal cancer exhibited loss of B2M but HLA-A expression was unaffected (**Figure 3D**). The lack of B2M expression in the tumor cells examined did not preclude the localization of HLA-A (human leukocyte antigen A) at the membrane, as indicated by the arrows in **Figure 3D**. Instances of HLA-A surface expression occurring independently of B2M have been documented in previous studies (23–28). The cumulative results of this part of our study generally concurred with previous reports describing incidence of HLA and B2M expression in diverse tumor types (29, 30). Additionally, there were examples that showed loss of HLA-A in cells positive for B2M, and vice versa, suggesting that the expression of these proteins was potentially independent from each other. Stroma was distinctly positive in every case (**Figures 3A–D**), as well as infiltrating immune cells (not shown).

**Figure 4:**
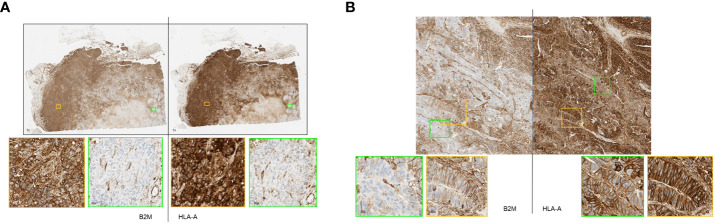
**(A, B)** Primary tumor stained by immunohistochemistry method showing remarkable heterogeneity of B2M and HLA-A expression. **(A)** Representative breast cancer case with heterogeneous expression of HLA-A and B2M. Strongly B2M positive (orange box) and B2M negative (green box) tumor areas adjacent to each other. Similarly, areas of strong HLA-A positivity and negativity were seen (orange and green box respectively). **(B)** Representative case of colorectal cancer displaying heterogeneous B2M expression with tumor areas showing strong membrane expression of B2M (orange box) adjacent to virtually negative areas (green box). HLA-A expression in the specimen was consistently positive on tumor cell membrane as well as cytoplasm (orange box and green box).

Some of these samples had highly heterogeneous expressions of the two proteins within the specimen. **Figure 4A** shows a section of a breast tumor having areas that displayed a complete loss of HLA-A and B2M (green boxes) alongside other areas that exhibited very high expression of both proteins (orange boxes). Visually, the expression of these proteins correlated well with each other as they either were clearly positive or clearly negative for both proteins concurrently. Other cases of intratumoral heterogeneity of expression were observed affecting only B2M or HLA-A. **Figure 4B** shows a colorectal cancer case with variable B2M expression ranging from clearly positive to negative with consistently positive expression of HLA-A throughout the whole section.

Overall, we stained B2M and HLA-A on a large set of primary (n= 420) and metastatic tumor samples (n=213 baseline and 116 matched on-treatment samples) of various histologies. The cut-off for definition of positive expression versus loss was set at a threshold of ≥50% tumor cells within a sample. Samples above this cut-off were classified as positive for B2M or HLA-A, respectively while cases with lower percentage positivity were classified as harboring a loss of the respective protein. Prior to establishing the cut-off at 50% we compared different, narrower thresholds and observed that the results were comparable regarding outcome and interpretation (not shown).

**Figure 5:**
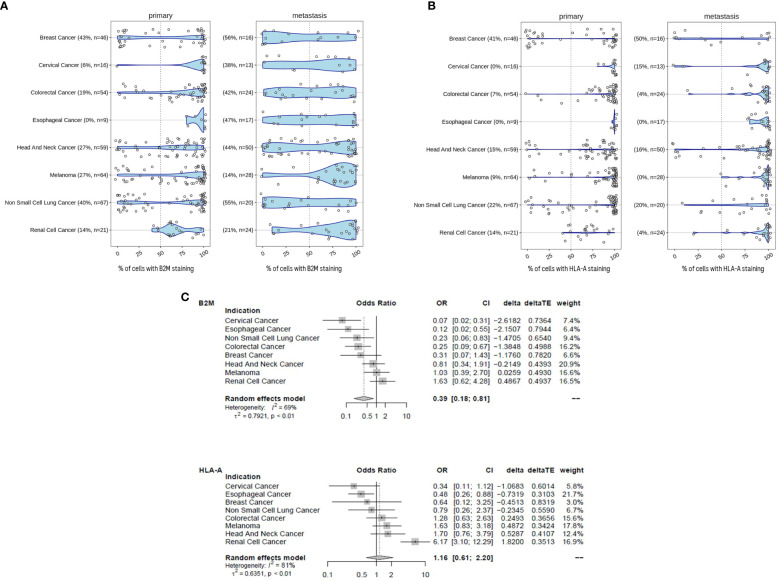
B2M loss is increased in metastatic disease. **(A)** B2M expression in primary and metastatic tumors determined by immunohistochemistry and assessed by certified pathologists. Tumors were determined to exhibit B2M loss when< 50% of tumor cells stained positive. We compared the distribution of the proportion of B2M-positive tumor cells between primary (n = 336) and metastatic samples (n = 182). Sample number tested and the percentage of samples with B2M loss are given in brackets for each tumor indication. Metastatic samples were more likely to be B2M-negative than primary tumor samples and the difference was statistically significant (logistic meta-regression, Odds ratio ~ 0.385, p-value ~ 0.0123). Statistics for each indication is given in **(C)**. **(B)** Similar to **(A)**, HLA-positive tumor cells were determined by immunohistochemistry. Tumors were determined to have HLA-A loss when< 50% of tumor cells stained positive. Sample number tested and the percentage of samples with HLA-A loss are given in brackets for each tumor indication. Statistics are provided in **(C)**. **(C)** Forest plot with the results of a meta-regression estimating the mean difference for B2M and HLAA loss in metastatic samples. Indications were coded as random effect. The figure shows the odds ratios with 95% confidence intervals and the relative weight of each indication for the overall effect. The average effect is given at the bottom, any shift to the left of an odds ratio of 1 is an increase in B2M and HLA-A loss, respectively.

For the following part of the study, the primary and metastatic tumors were not longitudinally matched in the same patient. The examination of B2M (**Figure 5A**) and HLA-A (**Figure 5B**) protein expression in tumors revealed a high degree of heterogeneity between patients. The extent of B2M loss varied considerably, depending on the tumor type and whether the sample was a primary tumor or distant metastasis. The prevalence of B2M loss in primary tumors ranged between 0% (esophageal cancer) and 43% (breast cancer) and between 14% (renal cancer) and 56% loss (breast cancer) in metastatic disease. The highest frequency of B2M loss was observed in metastatic breast cancer (56%) and metastatic NSCLC (55%). Increased loss of B2M expression in metastasis compared to primary tumors was observed in 7 out of 8 indications. Only melanoma samples displayed a relatively modest decrease in B2M loss, with 21% loss in primary to 14% loss in metastatic disease. Across all the tumor types, metastatic samples were approximately 2.5 times more likely to be B2M negative compared to primary tumor samples (Odds ratio ~ 0.39 [CI 0.18 - 0.81], p-value ~ 0.012). Statistics for individual indications and across indications are given in **Figure 5C**. Within these types of tumors, more frequent loss in metastatic samples was significant in the case of cervical cancer (p=0.001), colorectal cancer (p=0.006), esophageal cancer (p=0.027) and NSCLC (p=0.023). Like B2M, HLA-A protein loss was also highly prevalent in tumors of different indications and across disease stages (**Figure 5B**). The prevalence of samples with HLA-A loss in primary tumors ranged between 0% (cervical cancer, esophageal cancer) and 41% (breast cancer) and between 0% (esophageal cancer and melanoma) and 50% loss (breast cancer) in metastatic disease. In contrast to B2M, there was no obvious trend for an altered frequency of HLA-A expression in metastatic diseases versus primary tumors (meta regression analysis Odds Ratio 1.16, CI 0.61-2.2, p-value ~ 0.65).

This data is in concordance with a previously published report (31) demonstrating that essential components of the MHC class I presentation complex are missing in a fraction of tumors. The increased occurrence of loss of expression in metastatic samples may be associated with progression to advanced stages of disease. Potential causes include an accumulation of loss over time, tumor evolution and emergence of resistant clones (32). This study further validates the previous indication-specific findings over a broad panel of diverse tumors and extends the comparison in early and metastatic disease.

We subsequently correlated the expression of these two proteins with histological and clinical response to immunotherapy. All of the subsequent experiments were performed with samples obtained during the course of early-phase clinical trials conducted by Roche. Details on the clinical studies from which the samples were derived are listed in **Table 1**.

### B2M expression is increased in CD8 inflamed tumors

**Figure 6:**
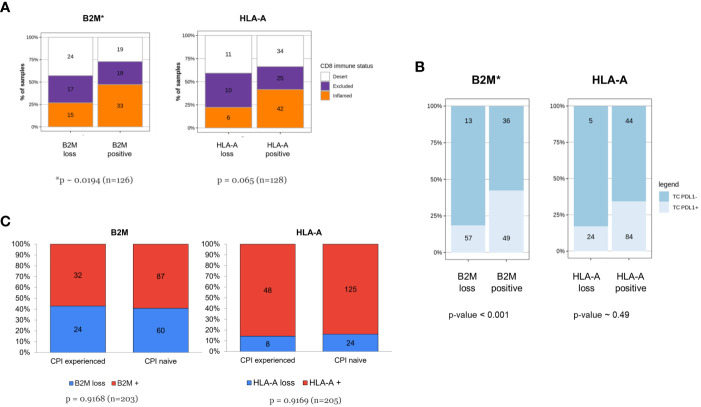
**(A-C)** Tumors of patients resistant to checkpoint inhibitor treatment are not enriched for HLA-A or B2M loss despite association of B2M expression with CD8 infiltration and PDL1 expression. **(A)** Increased frequency of CD8+ inflamed immune phenotype in tumors with B2M expression. The respective proportion of metastatic tumors with CD8+ inflamed, excluded or desert phenotype is compared between tumors displaying loss or positive HLA-A or B2M expression status. Tumors positive for B2M show a relative increase in proportion of CD8+ T-cell inflamed tumors (p< 0.05, χ^2^-test for equal proportions). **(B)** B2M positivity is associated with PDL1 expression. Distribution of tumor PDL1 expression in specimens which are either positive or negative for HLA-A and B2M, assessed at the time of enrolment in a Roche-sponsored clinical trial. Tumors positive for B2M were more likely to express PDL1 than those that were B2M negative and the difference was statistically significant (n = 155, p-value< 0.001, logistic regression adjusted for two clinical PDL1 staining procedures, see methods for details). **(C)** Tumors of patients resistant to prior checkpoint inhibitor treatment are not enriched for HLA-A or B2M loss. The figure illustrates distribution of HLA-A and B2M expression in tumors from patients who were CPI-experienced or CPI- naive at the time of the biopsy. CPI-experienced patients had advanced disease and had failed at least one prior line of checkpoint inhibitor treatment. They were therefore eligible for and enrolled in an early-phase 1 clinical trial, testing a new investigative treatment as monotherapy or as a combination. CPI-naive patients were patients with advanced disease who were enrolled in an early-phase clinical trial and consequently were not on standard-of-care treatment or had failed at least one prior line of non-CPI therapy. Difference in proportions was tested with a χ^2^-test and sample numbers are given for each group.

In 2017, Chen and Mellman (33) described three cancer immune phenotypes; “immune deserted”, “immune-excluded” and inflamed, based on specific underlying biological mechanisms. Others (17, 34) used the spatial distribution of tumor-infiltrating T cells for defining the same three immuno-phenotypes. Like those approaches, we categorized tumors based on the spatial distribution of CD8+cells and examined the association between these immune infiltration phenotypes and HLA-A or B2M expression. B2M expression or loss was not uniformly dispersed among CD8+ immune phenotypes (**Figure 6A**). Tumors classified as CD8+ “deserts” were more frequently associated with B2M loss, while CD8+ “inflamed” tumors were more frequently associated with detectable B2M expression (chi-squared test for independence p<0.02, n=126). A trend that did not reach statistical significance was noted for HLA-A and association with CD8+ immune phenotypes (chi-squared test for independence p<0.07, n=127). Our findings are in line with previously published data on the association of HLA or B2M loss with decreased CD8 infiltration (35, 36).

### Baseline tumor samples expressing PD-L1 are more likely to exhibit B2M positivity

Next, we correlated the relative expression of B2M with PD-L1 assessed by IHC (**Figure 6B**) in longitudinal samples from patients. Since the tumor PDL1 status was determined using either with SP142 or SP263 antibody clones, a covariate was added to enable testing for significant association between PDL1 positive staining and likelihood for HLA-A or B2M positive cells (details in M&M section). Paired tumor samples were baseline specimens obtained before start of treatment paired with specimens during treatment. At baseline, tumors categorized as positive for PD-L1 expression (TC+) were significantly enriched for positive B2M expression (log-Odds ~1.5, p~0.001, n=155) which did not carry over to on-treatment samples, where no apparent differences were detectable (not shown). No association between HLA-A and PD-L1 was identified at baseline (log-Odds ~0.275, p-value ~ 0.49, n=157) or after treatment (not shown), possibly due to higher overall expression of HLA-A and by comparison low frequency of loss.

### B2M/HLA-A expression distribution was not affected by prior CPI therapy

In our cohort of clinical samples collected at baseline, we also investigated the effect of prior CPI therapy on B2M and HLA-A loss. The distribution of expression loss in samples derived from CPI experienced patients (patients who had received prior CPI therapy, n=56) was compared to samples from CPI therapy-naive patients (n=149). CPI-experienced patients were defined as patients who had experienced treatment failure (patients with either primary or acquired resistance to CPI therapy) and who had subsequently enrolled in one of the clinical studies sponsored by Roche listed in **Table 1**. Samples originating from these patients were collected at baseline before commencing the new treatment investigated by the Roche sponsored trial, after CPI therapy failure in a previous trial. CPI therapy-naive patients did not receive CPI in a previous trial, but likely have received other non-CPI, standard-of-care treatments. As observed in **Figure 6C**, the distribution of loss was largely comparable in CPI-experienced and CPI-naive patients. This observation is inconsistent with the hypothesis that MHC class I downregulation is a critical immune evasion and CPI resistance mechanism since we did not see a higher frequency of MHC class I loss in patients treated previously with CPI and who subsequently progressed compared to the CPI-naive patient population.

### High B2M expression within on-treatment samples (trend for HLA-A) is positively associated with outcome (RECIST), while baseline expression is not predictive

**Figure 7:**
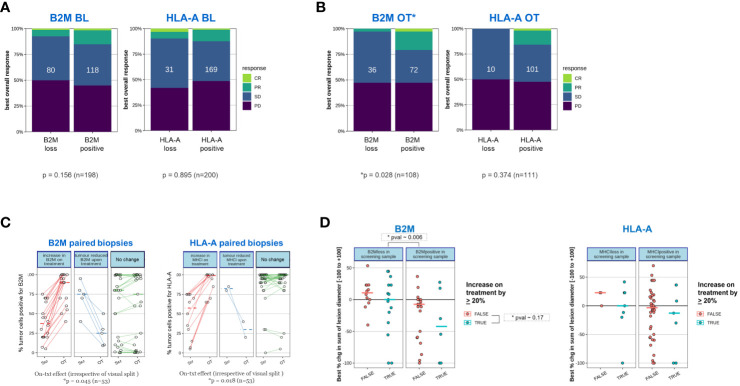
Tumors of patients who responded to a novel immunotherapy regimen are more likely to express B2M after initiation of treatment. The proportion of clinically confirmed responses in patients according to RECIST 1.1 were plotted against expression status of B2M and HLA as determined before initiation of treatment (BL) or after treatment initiation (on-treatment, OT). On-treatment samples were derived from patients after receiving either Simlukafusp alfa (FAP IL2v), Cergutuzumab (CEA IL2v) or Selicrelumab (anti-CD40) with or without Atezolizumab. For details, please refer to the Materials and Methods section. Responses were assessed on target lesions according to RECIST 1.1: PD progressive disease, SD stable disease, PR partial response, CR: complete response. Difference in proportions was tested with a χ^2^-test and sample numbers are given in each box. **(A)** Proportion of clinically confirmed responses in patients according to RECIST 1.1 corresponding to expression status of B2M and HLA-A before initiation of treatment. **(B)** As in **(A)**, proportion of clinically confirmed responses in patients according to RECIST 1.1 corresponding to expression status of B2M and HLA after initiation of treatment (on-treatment). On-treatment biopsies were scheduled after the second or third treatment cycle. **(C)** Tumors with B2M and HLA-A loss before treatment frequently upregulate B2M and HLA-A expression after treatment initiation. We compared B2M and HLA-A expression in cases where paired biopsies obtained at enrolment and on-treatment were available. Among samples that displayed B2M or HLA-A loss at enrolment, most on-treatment biopsies showed a substantial increase in B2M and HLA-A, respectively. Significance was tested with a paired, one-sided Student’s t-test after logit transformation to obtain equal variance. The plot visually splits samples with an on-treatment increase of more than 20% (left panel; red lines) from those with an on-treatment decrease of more than 20% (middle panel; blue lines) or no substantial change (right panel, green lines) for convenience of visualization. The statistical test was performed on the whole group of patients for B2M and HLA-A, respectively. **(D)** Patients with treatment-induced upregulation of B2M or HLA-A show a tendency toward a larger decrease in tumor target lesion size compared to patients without increase in expression. Tumor lesions were correlated for changes in tumor size and B2M or HLA-A expression. Associations were assessed before initiation of treatment as well as presence or absence of a significant increase in tumor size after treatment. An arbitrary threshold of ≥20% increase in positive tumor cells was set as the criterion for on-treatment increase in expression of the two proteins. Color coding was as follows: red- false=no increase observed; blue- true=increase was observed. Change in tumor size was measured using the established clinical parameter greatest percent change in sum of lesion diameters (SLD), which is the greatest decrease in tumor lesion size observed at any measurement during the time each patient remained on study. Negative SLD values indicate actual tumor shrinkage. Due to the relatively small sample sizes in the subgroup analysis, there is insufficient power to establish statistical significance. Regardless of B2M expression change, the presence of B2M protein at baseline was significantly associated with a larger decrease in SLD (p=0.006).

We then evaluated whether the frequency of response to treatment (defined as best response according to RECIST 1.1 (30)) correlated to presence or absence of B2M and HLA-A expression. It may be speculated that if loss of expression represents a relevant tumor escape mechanism leading to immunotherapy resistance, a correlation between protein expression and clinical responses would be noted. Indeed, **Figure 7A** shows a trend for greater clinical benefit (aggregated partial response, PR; complete response, CR) in patients expressing B2M and HLA, indicative of a functional MHC I presentation pathway being relevant for the respective immunotherapies tested. However, this trend did not reach statistical significance. What is noteworthy is that the association of clinical benefit with B2M and HLA expression was strengthened when the on-treatment expression levels were used in the analyses (**Figure 7B**). The positive association of on-treatment B2M expression with clinical outcome was significant (Chi-squared test of independence, p=0.028, n=108), while a clear but non-significant trend was observed for on-treatment HLA-A (Chi-squared test of independence, p=0.374, n=111), presumably due to the overall low frequency of HLA-A loss cases obtained in this subgroup analysis.

### B2M and HLA-A expression is frequently increased on-treatment

The increased association of expression of MHC class I components with clinical benefit, specifically for on-treatment samples led us to investigate whether the expression of HLA-A and B2M was more dynamic and influenced through treatment than recognized previously. In **Figure 7C**, the expression status of B2M and HLA in paired biopsies was assessed before and on treatment. In this analysis, only cases with available longitudinally matched baseline and on-treatment samples from the same patient were included to reduce any potential for bias. For better visual separation, longitudinal samples with or without a minimum change of 20% in expression on tumor cells in either direction were highlighted. Irrespective of the visual separation, the average expression of both B2M and HLA-A was significantly higher on treatment compared to the baseline time point (B2M p~0.045, HLA-A p ~0.018, n=53, one sided t-test), confirming a frequent increase in expression of both proteins on tumor cells.

### Patients with treatment-induced HLA-A or B2M upregulation show a trend towards higher tumor shrinkage based on best percentage change in sum of lesion diameter

We then examined whether patients with treatment-induced upregulation of B2M or HLA-A expression had a higher likelihood to experience clinical benefit. For this investigation, change in tumor size was investigated using the established clinical parameter greatest percent change in sum of lesion diameters (SLD). SLD is a continuous variable for measurement of tumor shrinkage underlying the categorical RECIST criteria which applies predefined cut-offs (31). We separated patients into distinct categories based on presence or absence of B2M or HLA-A at baseline and further stratified if upregulation of HLA-A or B2M in the on-treatment sample was observed, applying a cut-off increase by >20% relative to baseline. We noted regardless of B2M expression change, the presence of B2M protein at baseline was significantly associated with a larger decrease in SLD (p~0.006) compared to baseline B2M-negative tumors. This finding is not in line with our data showing lack of statistically significant B2M baseline association with outcome according to RECIST criteria. This discrepancy can be explained by the higher sensitivity of SLD in detecting smaller or transient changes in tumor lesion size compared to RECIST. (**Figure 7D**). A trend was observed for a higher decrease in the sum of lesion diameter (SLD) in patients with upregulated expression for both B2M and HLA-A, regardless of baseline expression status. However, this trend was not significant, presumably due to the overall low frequency of cases in these two subgroup analyses.

### Triple biomarker combination indicates an improvement in sensitivity and specificity over single or dual markers for response prediction to immunotherapies

**Figure 8:**
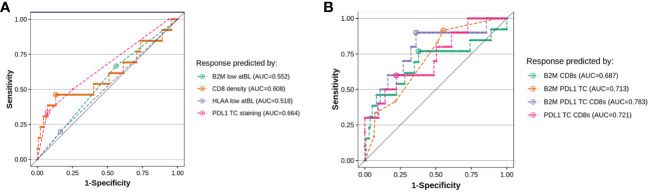
Evaluating B2M status at baseline in addition to T-cell infiltration improves the accuracy of predictive scoring for responders. We evaluated the value of testing for B2M status at baseline in addition to either CD8 density or PDL1 staining on baseline tumor biopsies, by fitting a logistic regression on all evaluable patients in our dataset (10-15 responders and 121-150 non-responders, depending on the combination of markers, responders defined as RECIST 1.1 partial or complete response). The plots show the comparison of the sensitivity versus specificity of the single predictor models **(A)** and each pairwise combination of B2M, PDL1 TC and CD8 density **(B)**. The dotted line visualizes random chance, the round dot in each curve indicates the point of optimal accuracy for each model (Youden’s index).

CD8 infiltration density and PD-L1 expression at baseline are known prognostic and/or predictive biomarkers (37). While our results do not substantiate either baseline HLA-A or B2M as individual markers for prognostic or predictive purposes, we investigated whether a score combining CD8, PD-L1 and baseline B2M (or HLA-A) would improve on predicting efficacy compared to single predictive biomarkers. **Figure 8A** shows a receiver-operating-curve (ROC) for the individual markers applied to the clinical samples and associated clinical benefit data, using RECIST criteria. The analysis confirmed that tumor PD-L1 and CD8 density have the highest predictive power for response, with an area-under-the-curve (AUC) of 0.664 and 0.608 (a value of 0.5 indicates complete absence of predictive performance). When evaluating combinations (**Figure 8B**), both CD8 density and B2M staining added to PD-L1 in performance. A triplet score (PD-L1, CD8 infiltration and B2M expression) reached an AUC of ~0.78, or a sensitivity of 64% and a specificity of 90% (n=121), though the relative scarcity of responders meant that it could not be unequivocally established whether a triple combination was significantly better as predictive score. In all comparisons, RCC patients had to be excluded because increased CD8 infiltration was an unfavorable predictor of response to treatment in this disease indication. This is in line with published data (38), showing CD8+ cells are an unfavorable prognostic biomarker only in RCC.

## Discussion

The primary goal of our study was to examine how the loss of tumor MHC I antigen presentation affects the efficacy of novel immunotherapeutic drugs, not just checkpoint inhibitors (CPI) but also therapies aimed at enhancing CD8 T cell-mediated killing. We hypothesized that patients lacking functional MHC I expression might not benefit from such treatments, as CD8 T cells require MHC I to recognize cancer cells. Confirming this hypothesis could lead to the exclusion of MHC I deficient patients from clinical trials: firstly, because these patients wouldn’t benefit from treatments targeting CD8 T cell activation, and secondly, to avoid false negatives in early efficacy assessments crucial for advancing a drug into further trial phases.

However, our findings suggest caution against such exclusion. The baseline measurement of HLA-A and B2M, our tested tissue markers, indicated that excluding patients based on these markers would eliminate many who could benefit from these therapies. To ensure our conclusions weren’t skewed by the inclusion of patients receiving CPIs as part of combination therapies, we conducted a separate analysis, excluding 55 patients at baseline and 27 on treatment who received approved CPIs. The results (data not shown), consistent with those in **Figures 7A, B**, reinforce that our findings apply broadly to immunotherapies that primarily activate T cells, not just to CPI treatments.

In our study, we have identified a significant correlation between the upregulation of B2M during immunotherapy and improved clinical outcomes, which underscores its potential as a more effective biomarker compared to HLA-A. B2M, a vital component of the major histocompatibility complex (MHC) class I molecules, is crucial in antigen presentation to CD8+ T cells and in modulating the immune response against tumor cells. B2M and HLA loss in tumor cells has been extensively studied from a molecular and mechanistic perspective and its potential implications for cancer immunotherapy (reviewed in (3)). The principal aim of our study was to explore whether B2M and HLA had utility as a predictive biomarker in early-phase cancer immunotherapy trials. We established a robust, validated, semi-automated method of IHC staining for paraffin-embedded tumor tissue that could potentially be utilized as a Companion Diagnostic tool for patient selection. Despite significant antibody screening efforts, including generation of *de novo* monoclonal antibodies (data not shown), the only HLA-specific antibody meeting the quality standards required for prospective patient selection on FFPE tissue was EP3195Y, which reacted to HLA-A instead of recognizing pan-HLA. Due to their close co-location on chromosome 6, it has been previously suggested that HLA-A,-B and -C genes are often concurrently lost through a combination of hard loss and through epigenetic mechanisms (21, 22), however we do acknowledge that this co-occurrence is not universally true. Hence, our results are in part derived from focusing on HLA-A, and this approach may have constrained our understanding of the complete immune response. While our study sheds light on the limitations of using HLA-A alone, it also highlights the potential of B2M, especially in the context of therapies targeting immune checkpoints such as PD-1/PD-L1 inhibitors. B2M’s role in reflecting the heightened immune activity during treatment makes it a more encompassing and reliable biomarker than HLA-A. This insight underscores the importance of considering a comprehensive approach that includes both individual HLA molecules and broader markers like B2M for a more accurate evaluation of immunotherapeutic efficacy in cancer treatment.

A limitation in our study is that the IHC-based assessment is oblivious to individual somatic mutations or loss on a specific HLA allele and to resulting alterations of the repertoire of tumor-antigens presented by MHC I protein complexes. Montesion et al. (39) examined a large clinico-genomics database including patients treated with CPIs for loss of HLA-A and described loss of the allele known to present neoantigens from driver mutations in KRAS and TP53, suggestive of selective pressure leading to specific LOH. Single HLA allele loss has originally been described in immune-infiltrated lung tumors with high neoantigen load in the TRACER-X cohort (40).Yet, occurrence of HLA-LOH was not predictive of response failure (defined by RECIST) in a meta-analysis of 7 tumor types (41), and even in presence of HLA-LOH, sustained expression of MHC I protein complexes would increase presentation of neoantigens via the remaining alleles. The assessment of single allele (LOH) versus complete loss by broadly accepted algorithms in our sample set was prohibited by lack of normal tissue control samples. Based on an analysis performed on a subset of the samples in this study, LOH (including both loss of expression as well as function) frequency was estimated to be relatively low (<10%), suggesting LOH would only represent a minor potential mechanism of resistance (data not shown) and usually on one allele only, excluding hard loss as a dominant mechanism. Our results are in line with a previous study that has reported B2M functional mutations/deletions only in 1.4% of cases (41). Nevertheless, specific alterations of MHC I alleles or changes of the peptide repertoire presented in tumors globally positive for MHC I as assessed by IHC requires further investigation.

The results of our IHC staining demonstrated that HLA-A and B2M protein loss occurred between 0-56% in diverse tumor types and subtypes with a higher frequency of B2M and HLA-A loss in metastatic specimens. Transcriptional or translational downregulation may be epigenetic but can be overcome in a large fraction of patients, as our results indicated that B2M and HLA-A expression increased during immunotherapy in a substantial fraction of specimens that had low or no expression of these proteins at baseline. Cumulatively, our results underscore that loss of B2M and HLA-A expression is specific to tumor cells but in the majority of cases it is not permanent and can be upregulated through immunotherapy treatment. Interferon-γ related pathways and interferon responsive elements (42) may be responsible for the upregulation and the phenomenon is specific for tumor cells since normal stroma or adjacent non-malignant tissue remain unchanged.

In our studies, only B2M and not HLA-A expression in tumor biopsies taken during ongoing immunotherapy correlated with clinical benefit, while tumor HLA-A or B2M measured before start of therapy (baseline samples) did not correlate to subsequent clinical outcome. Dhatchinamoorthy K. et al. (3) describes a “chicken and egg” paradigm whereby the cause-effect relationship between HLA-A/B2M expression and effector T cells becomes indistinguishable. Activated T cells produce cytokines and inflammatory factors that stimulate HLA-A and B2M expression. However, the expression of these molecules is a prerequisite to tumor recognition by T cells. Given that on-treatment and not baseline B2M expression was the only factor that correlated to clinical outcome in our studies, we speculate that the immunotherapy induces an immune activation and local inflammation that further drive MHC I upregulation and subsequently lead to clinical response.

We have also investigated combinations of pretreatment B2M and HLA levels relative to tumor PD-L1 expression and CD8+ tumor infiltration, which are known biomarkers for response to checkpoint inhibitor therapy (37, 38, 43). The triple combination of tumor PD-L1, intratumoral CD8+ cells and pretreatment B2M expression improved the prediction score over the single marker or dual combination of tumor PD-L1 and intratumoral CD8+ cell infiltration. We are therefore of the opinion that while HLA-A and B2M may not be independently useful as biomarkers for patient selection, they may have utility within a panel of potential biomarkers used for patient selection in immunotherapy clinical trials.

There are a few key observations regarding HLA-A and B2M that can be made from our studies. In line with previous reports, HLA-A and B2M low tumors had less CD8+ T cell infiltrate (31, 35). However, B2M expression was increased in CD8 high, PD-L1+ tumors which suggest that functional anergy induced through PD-1 on T-cells interacting with tumor PD-L1 may be a more dominant pathway of immune escape and B2M downregulation may be of lesser consequence in its presence. Secondly, B2M is frequently upregulated in tumors following immunotherapy which reflects immune activation and resultant inflammatory cytokines and soluble factors that in turn may upregulate B2M expression. Thirdly, the frequency of HLA-A and B2M loss in tumors of patients who had previously received immunotherapy was comparable and not statistically different from immunotherapy-naïve patients. Our results indicate that B2M downregulation may not be a universal and elemental escape mechanism for tumors refractory to immunotherapy and digress from other reports that describe loss of B2M as a direct cause of resistance to checkpoint inhibitor therapy (44–46). One explanation for our diverging results may be due to our studies being conducted on a diverse range of tumors while these reports linking B2M loss to immunotherapy resistance was done in melanoma and lung adenocarcinoma. Furthermore, our results indicate that usually the loss of B2M and HLA-A was not a “hard loss” and could be reversed once the cell-mediated immune system was restored by immunotherapy.

In conclusion, our results substantiate that B2M and HLA-A protein loss occurs at varying frequencies over different tumor types. Importantly, transcriptional or translational repression can be overcome by immunotherapy in a large fraction of patients. The treatment-induced upregulation of MHC I expression may be an important mechanism contributing to efficacy. A consequence of this finding is that protein expression at baseline is not predictive. B2M detection by IHC, in contrast to HLA, being insensitive to polymorphisms, is a universal biomarker indicative of a functional MHC class I presentation machinery and has therefore broad application potential. We speculate that changes in B2M levels in tumor biopsies taken early during immunotherapy may be an early indicator of subsequent clinical response.

## Data availability statement

The original contributions presented in the study are included in the article/**Supplementary Material**. Further inquiries can be directed to the corresponding author.

## Ethics statement

Ethical approval was not required for the studies involving humans because the manuscript describes research performed with left-over specimens from multiple clinical trials. Each individual clinical study had ethics approved by the local IRB at each participating study site, but there was no dedicated IRB review or approval of the specific research performed and described in the manuscript. However, the objective for using the left-over specimens for the research described in the manuscript was consistent with the content and overall objectives stated in the informed consent forms signed by the individual patients as well as with the protocol text approved by the ethics committees/IRBs. Also purchased material was used. The studies were conducted in accordance with the local legislation and institutional requirements. Written informed consent to participate in this study was not required from the participants or the participants’ legal guardians/next of kin in accordance with the national legislation and the institutional requirements.

## Author contributions

BR: Conceptualization, Investigation, Methodology, Supervision, Visualization, Writing – original draft, Writing – review & editing. JA: Data curation, Formal analysis, Methodology, Visualization, Writing – original draft, Writing – review & editing. SD: Conceptualization, Supervision, Writing – original draft, Writing – review & editing. NG: Data curation, Methodology, Writing – original draft, Writing – review & editing. AH: Data curation, Methodology, Writing – original draft. NR: Methodology, Supervision, Validation, Writing – original draft, Writing – review & editing. BG: Conceptualization, Writing – review & editing.

## Supplementary material

Supplementary Figure 1 Detailed Pre-treatment Profiles by Study in HLA-A and B2M Staining Cohort This figure illustrates the frequency of pre-treatments within our 213-patient cohort, categorized by the specific study each patient participated in (refer to study identifiers in ). The pre-treatment data, extracted from the Clinical Study Reports by Roche, encompasses only those treatments administered (i) before the initial dose as per the trial protocol, and (ii) recognized as either premedication or active cancer therapy. All treatments are named in alignment with the standardized terminology used by the Global Drug Safety Reporting (GDSR) and the Medical Dictionary for Regulatory Activities (MedDRA).
