# Supplementary figures and images for: Tumor beta2-microglobulin and HLA-A expression is increased by immunotherapy and can predict response to CIT in association with other biomarkers
Source: Front Immunol. 2024 Feb 22;15:1285049. doi: 10.3389/fimmu.2024.1285049 (PMC10917949; doi:10.3389/fimmu.2024.1285049)

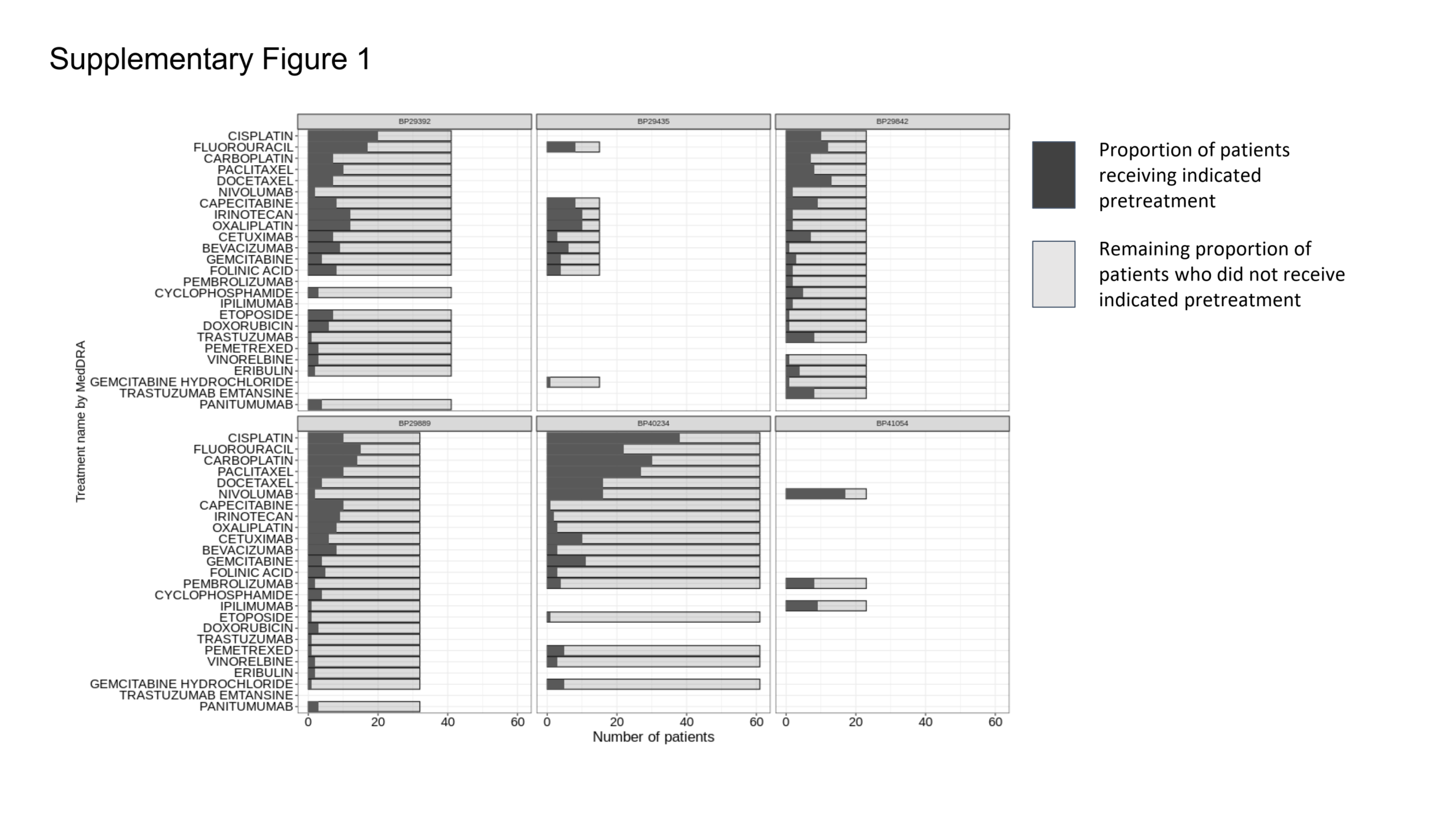

Supplement: Supplementary Figure 1 — Detailed Pre-treatment Profiles by Study in HLA-A and B2M Staining Cohort This figure illustrates the frequency of pre-treatments within our 213-patient cohort, categorized by the specific study each patient participated in (refer to study identifiers in ). The pre-treatment data, extracted from the Clinical Study Reports by Roche, encompasses only those treatments administered (i) before the initial dose as per the trial protocol, and (ii) recognized as either premedication or active cancer therapy. All treatments are named in alignment with the standardized terminology used by the Global Drug Safety Reporting (GDSR) and the Medical Dictionary for Regulatory Activities (MedDRA). [file Image_1.tif]
